# Endoplasmic Reticulum Stress Induces Macrophages to Produce IL-1β During *Mycobacterium bovis* Infection via a Positive Feedback Loop Between Mitochondrial Damage and Inflammasome Activation

**DOI:** 10.3389/fimmu.2019.00268

**Published:** 2019-02-21

**Authors:** Yi Liao, Tariq Hussain, Chunfa Liu, Yongyong Cui, Jie Wang, Jiao Yao, Hehua Chen, Yinjuan Song, Naveed Sabir, Mazhar Hussain, Deming Zhao, Xiangmei Zhou

**Affiliations:** ^1^Key Laboratory of Animal Epidemiology and Zoonosis, Ministry of Agriculture, National Animal Transmissible Spongiform Encephalopathy Laboratory, College of Veterinary Medicine, China Agricultural University, Beijing, China; ^2^National Center for Tuberculosis Control and Prevention, Chinese Center for Disease Control and Prevention, Beijing, China; ^3^Department of Microbiology and Immunology, Feinberg School of Medicine, Northwestern University, Chicago, IL, United States

**Keywords:** *Mycobacterium bovis*, ERS, mitochondrial damage, inflammasome, IL-1β

## Abstract

*Mycobacterium bovis*, the causative agent of tuberculosis in cattle and humans, infects host macrophages and induces endoplasmic reticulum stress (ERS), mitochondrial damage, and interleukin (IL)-1β production. The relationship between these phenotypes is yet to be elucidated. In this study, we investigated the role of ERS in mitochondrial damage and IL-1β production in macrophages during infection with a virulent *M. bovis* strain. We found that ERS activates the inflammasome via NOD-like receptor family, pyrin domain-containing 3 (NLRP3)-caspase-8 and that IFN-inducible protein absent in melanoma 2 (AIM2) triggered mitochondrial damage. ERS increased reactive oxygen species (ROS), which promoted translocation of the inflammasome to the mitochondria. NLRP3, but not AIM2, was involved in the ERS-induced cleavage of caspase-8 and Bid, leading to mitochondrial damage, which was required for the production of mature IL-1β. Our data suggest that ERS induces macrophages to produce mature IL-1β during infection with virulent *M. bovis* through a positive feedback loop between mitochondrial damage and inflammasome activation. To the best of our knowledge, this is the first evidence of the involvement of ERS and mitochondrial damage in inflammasome activation during *M. bovis* infection.

## Introduction

*Mycobacterium tuberculosis* (*Mtb*) is the primary causative agent of human tuberculosis and is found worldwide. *M. bovis*, a member of the *Mycobacterium* complex, causes tuberculosis in humans and a broad range of animal species. In humans, the host immune response induced by *M. bovis* infection resembles that induced by *Mtb* (1). *M. bovis* mainly infects and replicates within host macrophages, which are important effector cells in the regulation of the protective innate immune response to resist intracellular bacterial multiplication.

Interleukin (IL)-1β is one of the key proinflammatory cytokines that play a critical role in innate immune responses. Mice defective in IL-1R or IL-1β are more sensitive to mycobacterial infection and have an increased bacterial burden (2–4). The increase in susceptibility of IL-1R-deficient mice results from the recruitment of defective immune cells to the site of infection and a deficiency in the formation of proper granuloma (5–7); these changes indicate the vital significance of IL-1β signaling to fight against mycobacterial infection. Mature IL-1β is formed in the cytoplasm from the precursor of IL-1β, pro-IL-1β, which is a biologically inactive form of IL-1β. The maturation of pro-IL-1β is dependent on the activation of a multiprotein complex signaling mechanism, which is referred to as inflammasome activation (8, 9). The activated inflammasome cleaves pro-IL-1β by cysteinyl aspartate specific proteinase (caspase-1), an enzymatic reaction that is followed by the secretion of the mature active IL-1β into the extracellular matrix (10). The explicit mechanisms of inflammasome activation and IL-1β production during *M. bovis* infection are yet to be elucidated.

Mitochondrial damage or destabilization is potentially crucial for activation of the inflammasome by numerous impetus (11). Different types of cell stress result in damage of the mitochondria, which initiates the inflammasome activation. Consistently, the NLRP3 inflammasome is recruited to the mitochondria, allowing it to function as a mitochondrial check framework compared to the apoptosome. Moreover, inflammasome recruitment to the mitochondria, and activation intensifies mitochondrial destabilization or dysfunction. Thus, mitochondrial damage and inflammasome activation form a positive feedback loop, which amplifies cellular stress signals.

The endoplasmic reticulum (ER) is a complicated, delicate organelle capable of protein folding, calcium stockpiling, and lipid or carbohydrate metabolism (12). The ER is highly sensitive to perturbation. Diverse cellular stresses, such as microbial infection, destabilization of calcium homeostasis, or redox imbalance, cause ER stress (ERS), which is characterized by the accumulation of unfolded proteins in the ER lumen. The ER establishes a connection to the mitochondria through a specialized structure, which is often referred to as mitochondria-associated membranes (MAMs). Interestingly, in the context of diverse ER signals, MAMs play a crucial role in the modulation of mitochondrial shape, redox status, and permeability, a process that is profoundly involved in a many cellular mechanisms, from inflammasome activation to cell death (13–15). The ER may serve as a bridge between stress-inducing elements and mitochondrial damage associated with inflammasome activation.

Three pathways, inositol-requiring enzyme 1 (IRE1), protein kinase RNA (PKR)-like ER kinase (PERK), and activating transcription factor 6 (ATF6), induce ERS reactions (16, 17), a homeostatic signaling network that controls the physiological outcomes of the stressed cell (18). Toll-like receptors (TLR), which distinguish mycobacterial ligands, selectively trigger IRE1 (19). Virulent *M. bovis* strains interact with the ER and induce X-box binding protein 1 (XBP-1) mRNA splicing, a process suggestive of IRE1 activation (20). In addition, our previous studies suggest that infection with virulent *M. bovis* strains induce inflammasome activation and IL-1β secretion in macrophages (21, 22). Moreover, infection of macrophages by *M. bovis* leads to inflammasome-dependent mitochondrial damage (21). The early published data drove us to theorize that, upon *M. bovis* infection, ERS triggers a positive feedback loop between mitochondrial damage and inflammasome activation, resulting in IL-1β production, as described in the current study.

## Materials and Methods

### Animal Infection Model

Six-week-old female C57BL/6 mice were obtained from Vital River Laboratory Animal Technology Co., Ltd (Beijing, China). Twenty-eight mice were divided into four groups: (1) Control group (*n* = 7); (2) 4-PBA group (*n* = 7); (3) *M. bovis*-infected group (*n* = 7); and (4) 4-PBA + *M. bovis*-infected group (*n* = 7). The animals were challenged by intranasal (i.n.) route with 200 CFU of virulent *M. bovis*. Animals in the 4-PBA group and 4-PBA + *M. bovis* group were treated with 4-PBA solution in drinking water (18.6 mg/mouse/day) for 3–6 weeks. After 3–6 weeks of infection, the mice were sacrificed. The blood and organs were collected aseptically for further experiments. At the indicated times, the blood was centrifuged at 10,000 rpm for 5 min to separate the serum for ELISA analysis. The lung tissues were removed and preserved in 10% formalin for histopathology or homogenized in RIPA lysis buffer (R0010, Solarbio, Beijing, China) for western blotting experiments.

### Cell Culture

To prepare Bone marrow derived macrophages (BMDMs) for culture, the mice were sacrificed by cervical dislocation and immersed in 75% ethanol for 5 min. The bilateral tibia and femur were separated and rinsed in PBS to remove the soft tissue around the bones. The ends of the bones were cut and bone marrow was scoured by RPMI1640 (Hyclone, Logan, UT, USA) into a centrifuge tube and centrifuged at 1,000 rpm for 10 min. The supernatant was discarded and the precipitate was resuspended in RPMI 1640 supplemented with 10 ng/mL M-CSF (Pepro Tech, Rocky Hill, NJ, USA) and 10% fetal bovine serum (FBS) (Gibco, Grand Island, NY, USA). After the cells were counted, 1 × 10^6^ cells were seeded in a cell culture dish (Corning, New York, NY, USA) and cultured for 7 days. On Day 7, BMDMs were collected and seeded into cell culture plates (Corning, New York, NY, USA) for 12 h before the test experiments.

### Bacterial Culture and Infection

*M. bovis* was obtained from the China Institute of Veterinary Drug Control (CVCC, Beijing, China). The bacteria were cultured in 7H9 medium (BD Biosciences, New York, NY, USA) supplemented with albumin-dextrose-catalase (ADC) enrichment solution and 0.05% Tween 80 (Difco, Leeuwarden, Netherlands) to a mid-logarithmic phase at 37°C in a shaking incubator. For enumeration of total viable bacilli, 10-fold serial dilutions were prepared in sterilized PBS. Each dilution was transferred in triplicate to 7H11 agar plates (BD Biosciences, New York, NY, USA) supplemented with ADC enrichment solution and 0.05% Tween 80 and the number of CFUs was counted at 3–4 weeks after incubation.

For cell infection model, *M. bovis* was added to BMDMs at a MOI 10 and incubated for 2 h. The inoculum was then removed and the cells were washed with PBS and cultured in fresh media at 37°C in a CO_2_ incubator. The first 2 h of the incubation period is considered the phagocytosis time period for macrophages and counted as 0 h post-infection. Samples of infected cells were collected at 0, 6, 24, and 48 h post-infection. For inhibitor-pretreated samples, BMDMs were treated with 10 μM cyclosporin A, 5 mM 4-PBA, 5 mM NAC, 500 μM MitoTEMPOL, 20 μM belnacasan, or 50 μM z-IETD-fmk, separately, for 1 h prior to infection. For the positive control samples, the cells were treated with 25 mM etoposide or 10 mg/mL tunicamycin for 4 h, and the LPS+ATP samples were pretreated with LPS (200 ng/mL) overnight, after which 1 mM ATP was then added for 4 h. At the indicated times, the cells were washed with PBS and collected for the subsequent experiments.

### Reagents

Rabbit monoclonal anti-mouse BiP antibody (ab108615), rabbit polyclonal anti-mouse phospho-IRE1 antibody (ab48187), rabbit polyclonal anti-mouse tBid antibody (ab10640), rabbit monoclonal anti-mouse Fl caspase-8 antibody (ab108333), and MitoTEMPOL (ab144644) were purchased from Abcam (Cambridge, MA, USA). Rabbit polyclonal anti-mouse cytochrome c antibody (10993-1-AP), rabbit polyclonal anti-mouse TOM20 antibody (11802-1-AP), rabbit polyclonal anti-mouse AIM2 antibody (20590-1-AP), rabbit polyclonal anti-mouse Cl caspase-8 antibody (13423-1-AP), rabbit polyclonal anti-mouse Bid antibody (10988-1-AP), and mouse monoclonal anti-mouse VDAC antibody (66345-1-lg) were obtained from Proteintech (Wuhan, Hubei, China). Rabbit monoclonal anti-mouse NLRP3 antibody (15101) and rabbit polyclonal anti-mouse cleaved-IL-1β (63124) antibody were obtained from Cell Signaling Technology (Danvers, MA, USA). Goat polyclonal anti-mouse IL-1β antibody (AF-401-NA) was obtained from R&D Systems (Minneapolis, MN, USA). Goat anti-rabbit secondary antibody (ZB-5301) and rabbit anti-goat secondary antibody (ZB-2306) were obtained from Beijing ZSGB Biotechnology (Beijing, China). Tunicamycin (TM) was purchased from Fermentek Ltd (Jerusalem, Israel). 4-Phenyl butyric acid (4-PBA) (11323) was purchased from Cayman Chemical (Ann Arbor, MI, USA). Lipopolysaccharide (LPS) (L8880), adenosine triphosphate (ATP) (IA0590), etoposide (ET) (IE0270), and rabbit polyclonal anti-mouse β-actin antibody (RG000120) were purchased from Solarbio (Beijing, China). N-Acetyl-cysteine (NAC) (S0077) and cyclosporine A (CsA) (S1563) were obtained from Beyotime Biotechnology (Wuhan, Hubei, China). Belnacasan (VX-765) (S2228) and z-IETD-fmk (S7314) were purchased from Selleckchem (Houston, TX, USA).

### Small Interfering RNA (siRNA) Transfection

Mouse NLRP3-targeting and AIM2-targeting siRNA oligonucleotides were obtained from Dharmacon (Lafayette, CO, USA). Mouse Bid-targeting siRNA oligonucleotide was obtained from Solarbio (Beijing, China). The sequences of siRNAs used in this study are listed in Table 1. For siRNA transfection, the cells were seeded in 24-well plates at a density of 1 × 10^5^ cells/well. Fifty nanomolar siRNA oligonucleotides (50 nM) were transfected into BMDMs by using HiPerFect transfection Reagent (Qiagen, Valencia, CA, USA) in accordance with manufacturer's guidelines. After 24 h, the transfection medium was replaced with fresh medium. After 48 h, the cells were harvested for expression analysis or the subsequent experiments.

**Table 1:** SiRNA used in this study.

**SiRNA name and sequence**	**(5^**′**^-3^**′**^)**
• NLRP3 (target-1)	5′-GGUGAAAUGUACUUAAAUC-3′
• NLRP3 (target-2)	5′-GGAUGGGUUUGCUGGGAUA-3′
• NLRP3 (target-3)	5′-ACACACCUCUAUCUACGAA-3′
• NLRP3 (target-4)	5′-GAAGUGGACUGCGAGAGAU-3′
• AIM2 (target-1)	5′-ACAUAGACACUGAGGGUAU-3′
• AIM2 (target-2)	5′-UGUCUAAGGCUUGGGAUAU-3′
• AIM2 (target-3)	5′-CUACCUGAGGAUAGCAUUU-3′
• AIM2 (target-4)	5′-AGUACUAAGAAAUCAGUGA-3′
• Bid (target)	5′-GGAGAACGACAAGGCCAUGCUGAUA-3′

### Isolation of Mitochondrial and Cytoplasmic Fractions

Mitochondrial and cytoplasmic fractions were separated from whole BMDM cell lysates by using the Cell Mitochondria Isolation Kit (Beyotime, Shanghai, China) in accordance with manufacturer's instructions.

### Quantitative Real-Time Polymerase Chain Reaction (RT-PCR)

Total RNA extraction and cDNA synthesis were performed by using TRIzol Reagent (Invitrogen, Carlsbad, CA, USA) and RevertAid First Strand cDNA Synthesis Kit (Thermo Fisher Scientific, Waltham, MA, USA), respectively, in accordance with the manufacturer's guidelines. The genomic DNA Mini Preparation Kit (Beyotime, Shanghai, China) was used for mtDNA extraction from the mitochondrial fraction. Quantitative RT-PCR was performed by using the ViiA7 Fast Real-Time PCR Systems (ABI, New York, NY, USA) and SYBR Green Master Mix (Bio-Rad, Hercules, CA, USA). The quantitative RT-PCR data were calculated by the comparative CT method (2^−−ΔΔ*CT*^). β-Actin and 18S rRNA were used as the internal control for cDNA and mtDNA, respectively. The sequences of the primers used in this study are listed in Table 2. All samples were analyzed in triplicate.

**Table 2:** Primers used in this study.

**Primer name and sequence**	**(5^**′**^-3^**′**^)**
• β -actin (forward)	5′-CCTTCTGACCCATTCCCACC-3′
• β -actin (reverse)	5′-GCTTCTTTGCAGCTCCTTCG-3′
• IL-1β (forward)	5′-AGAGCATCCAGCTTCAAATC-3′
• IL-1β (reverse)	5′-TCATCTCGGAGCCTGTAGTG-3′
• NLRP3 (forward)	5′-ATGGTATGCCAGGAGGACAG-3′
• NLRP3 (reverse)	5′-ATGCTCCTTGACCAGTTGGA-3′
• Cytochrome-c oxidase (forward)	5′-GCCCCAGATATAGCATTCCC-3′
• Cytochrome-c oxidase (reverse)	5′-GTTCATCCTGTTCCTGCTCC-3′
• 18S rRNA (forward)	5′-TAGAGGGACAAGTGGCGTTC-3′
• 18S rRNA (reverse)	5′-CGCTGAGCCAGTCAGTGT-3′

### Western Blotting

The cell culture supernatant proteins were extracted as described previously (21) and the total cell protein extraction was performed by using the Fast Protein Precipitation and Concentration Kit (Boster Biotech, Wuhan, China) in accordance with the manufacturer's instructions. Extracted proteins were mixed with 5 × SDS sample buffer and boiled for 10 min. The proteins were separated by 8–15% SDS-PAGE, followed by electrotransfer onto an Immobilon-P Transfer Membrane (Millipore, Billerica, MA, USA). The membranes were incubated with primary antibodies at 4°C overnight, washed with TBS-Tween solution for 30 min, and incubated with corresponding HRP-conjugated secondary antibodies for 1 h at 37°C. The blots were probed with an enhanced chemiluminescence detection system (Bio-Rad, Hercules, CA, USA).

### Enzyme-Linked Immunosorbent Assay (ELISA)

The concentration of IL-1β, IL-6, and TNF-α in cell supernatant or blood serum samples was measured by using ELISA kits from Cusabio (Wuhan, Hubei, China) and Neobioscience (Shenzhen, Guangdong, China). All ELISA assays were performed in accordance with the manufacturer's protocol.

### Transmission Electron Microscopy

The infection of BMDMs with *M. bovis* was performed as described above. After 48 h of infection, the cells were scraped and centrifuged for 5 min at 1,000 g. The section process and observation were carried out as described previously (21).

### Reactive Oxygen Species (ROS) Measurements

Measurements of ROS were performed by using the Reactive Oxygen Species Assay Kit according to the manufacturer's protocol (Beyotime, Shanghai, China). The cells were washed with PBS and incubated with DCFHDA at 37°C for 20 min. After incubation, the mean fluorescence intensity of 10,000 cells was evaluated by using flow cytometry at excitation and emission wavelengths of 488 nm and 535 nm, respectively. The statistical analysis for the data of mean fluorescence intensity was performed by using GraphPad Prism 6 software.

### Histological Analysis

The lung samples were embedded in paraffin and 4 μm sections were cut and then stained by using with hematoxylin-eosin (H&E) or Acid-Fast Stain. After numbering, the slides were analyzed for pathological changes. The lesion area was scored as: 0 = no lesion; 1 = lesion area accounts for 10% of the whole tissue; 2 = lesion area accounts for 20% of the whole tissue; 3 = lesion area accounts for 30% of the whole tissue; 4 = lesion area accounts for 40% of the whole tissue; 5 = lesion area accounts for 50% of the whole tissue; 6 = lesion area accounts for 60% of the whole tissue; 7 = lesion area accounts for 70% of the whole tissue; 8 = lesion area accounts for 80% of the whole tissue; 9 = lesion area accounts for 90% of the whole tissue; and 10 = lesion area accounts for 100% of the whole tissue.

### Cell Viability and Phagocytic Ability of Macrophages

BMDMs were seeded in 96 wells plate at a density of 4 × 10^5^ cells per well and pretreated with 4-PBA, NAC, CsA, MitoTEMPOL, siCon, siNLRP3, siAIM2, siBid, Belnacasan, and z-IETD-fmk in accordance with the protocol mentioned above. To evaluate the phagocytic ability of macrophages, the cells were washed with warm PBS and 4 × 10^6^ cells were added to each well. After incubation of the cells for 2 h with *M. bovis*, the inoculum was removed and the cells were washed with warm PBS and lysed for 10 min in a shaker at 37°C; subsequently, 100 μL of serial dilutions of the cells were plated separately on Middlebrook 7H11 agar plates and incubated at 37°C for 3–4 weeks. Colony counts were performed in triplicate. For cell viability analysis, the assay was performed by using the CellTiter 96 Aqueous One Solution Cell Proliferation Assay Kit (Promega, Madison, USA) in accordance with the manufacturer's guidelines.

### Statistical Analysis

Statistical analyses were computed by using Excel or GraphPad Prism 6 software. *P*-values of < 0.05 were considered representative of significant change. Student's *t*-test was used to compare two groups, one-way ANOVA followed by *post-hoc* Tukey's test for the comparison of multiple groups. The data shown in the manuscript represent three independent experiments. The error bars indicate the SD.

## Results

### *M. bovis*-induced Inflammasome Activation Requires ERS

**Figure 1:**
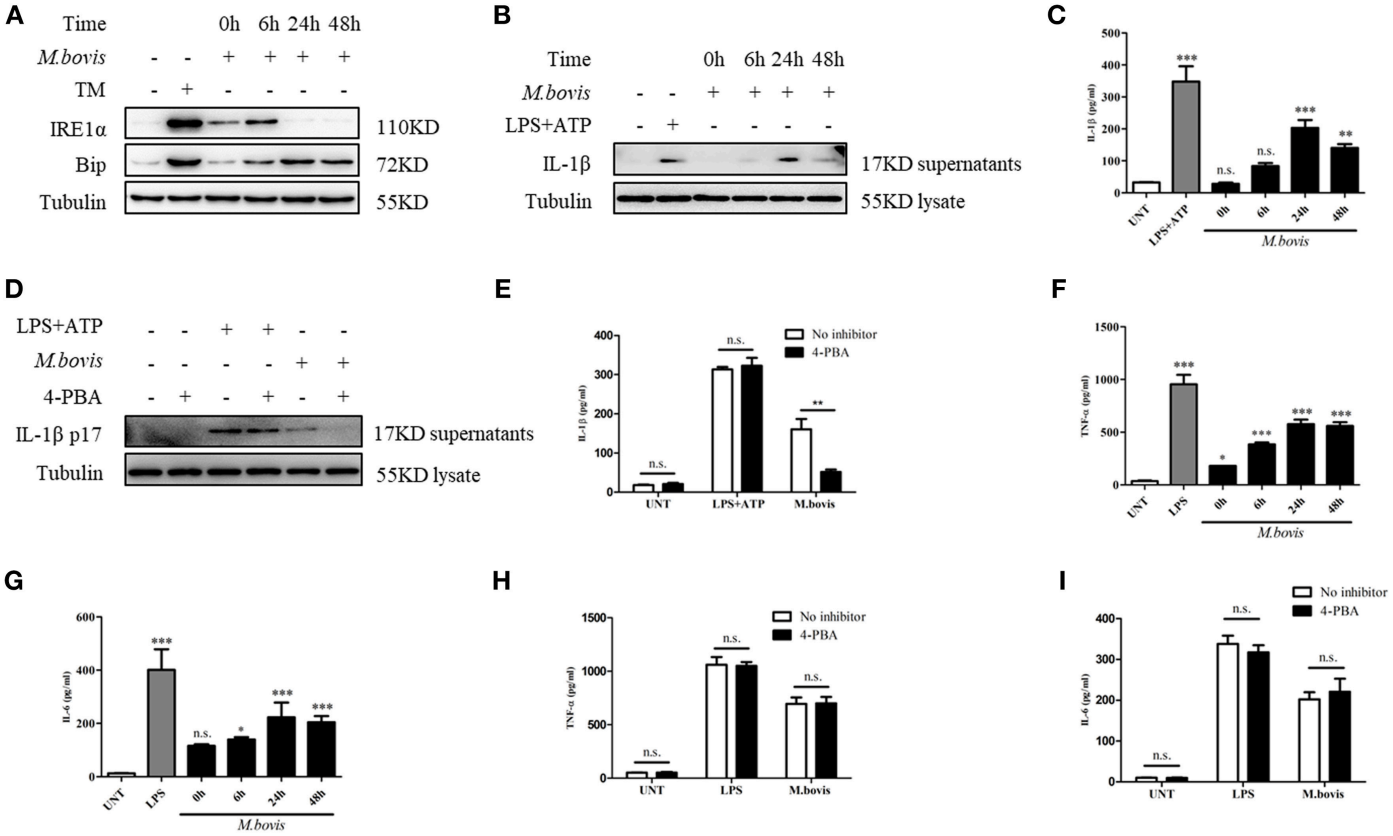
*M. bovis-*induced inflammasome activation requires ERS. **(A)** Immunoblot analysis at different time-points of Bip and p-IRE1α in lysates of BMDMs infected with *M. bovis* (MOI 10). **(B)** Immunoblot analysis at different time-points of IL-1β in supernatants of BMDMs infected with *M. bovis* (MOI 10). **(C)** ELISA for IL-1β analysis from supernatants of BMDMs infected with *M. bovis* (MOI 10). **(D)** IL-1β immunoblot analysis of supernatants from BMDM infected for 24 h with *M. bovis* (MOI 10) in the presence or absence of 4-PBA. **(E)** ELISA for IL-1β quantification from supernatants of BMDMs treated with or without 4-PBA for 1 h and then infected with *M. bovis* (MOI 10) for 24 h. ELISA for **(F)** TNF- α and **(G)** IL-6 detection in supernatants from M. bovis (MOI 10) infected BMDMs. ELISA for **(H)** TNF-α and **(I)** IL-6 detection from supernatants of BMDMs treated with or without 4-PBA for 1 h and then infected with *M. bovis* (MOI 10) for 24 h. TM, tunicamycin, positive control for ERS induced mitochondrial damage, 10 μg/mL; LPS+ATP, positive control for inflammasome activation, 200 ng/mL, and 1 mM, respectively; LPS, positive control for TNF-α and IL-6 production, 200 ng/mL; UNT, untreated; 4-PBA, 4-phenyl butyric acid, ERS inhibitor, 5 mM; MOI, multiplicity of infection. For **(C,F,G)**, Data are representative of at least three independent experiments, each performed in triplicate for WB. The results are shown are the mean ± SD. The asterisks indicate statistically significant differences compared with untreated cells (^*^P < 0.05, ^**^P < 0.01, ^***^P < 0.001, n.s., not significant). *P*-values were obtained by using one-way ANOVA followed by *post-hoc* Turkey' comparison test. For **(E,H,I)**, data are representative of at least three independent experiments. The results are shown as the mean ± SD. ^*^P < 0.05, ^**^P < 0.01, ^***^P < 0.001, n.s., not significant. *P*-values were analyzed using Student's *t*-test.

*Mtb* expresses multiple ligands that bind to members of the TLR family and triggers TLR signaling, which, in turn, induces ERS in macrophages (19). To determine whether *M. bovis* induced ERS in BMDM, we examined the protein expression of ERS markers. TM, an inducer of ERS, served as a positive control. The increased expression of Bip and phosphorylated IRE1α was observed in *M. bovis*-infected BMDMs (Figure 1A), which indicated that *M. bovis* infection induced ERS, similar to reports for other pathogens (23).

Previous studies have shown that ERS is able to provoke inflammasome activation (23, 24). It is known that *M. bovis* triggers IL-1β production (21, 22). To investigate whether ERS contributed to the production of IL-1β during *M. bovis* infection, we pretreated BMDMs with the chaperone 4-PBA, an ERS inhibitor, followed by infection with *M. bovis*. LPS+ATP, an agonist of the inflammasome, served as the positive control. The inhibition of ERS resulted in the reduction of IL-1β secretion in *M. bovis*-infected BMDMs, but not in BMDMs treated with LPS+ATP (Figures 1B–E). In addition, 4-PBA treatment had no significant effect on the secretion of TNF-α and IL-6 in response to LPS or *M. bovis* infection (Figures 1F–I). Collectively, these results indicated that ERS played a key role in the production of IL-1β during *M. bovis* infection.

### ERS-Induced Mitochondrial Dysfunction Mediated by Inflammasome Activation

**Figure 2:**
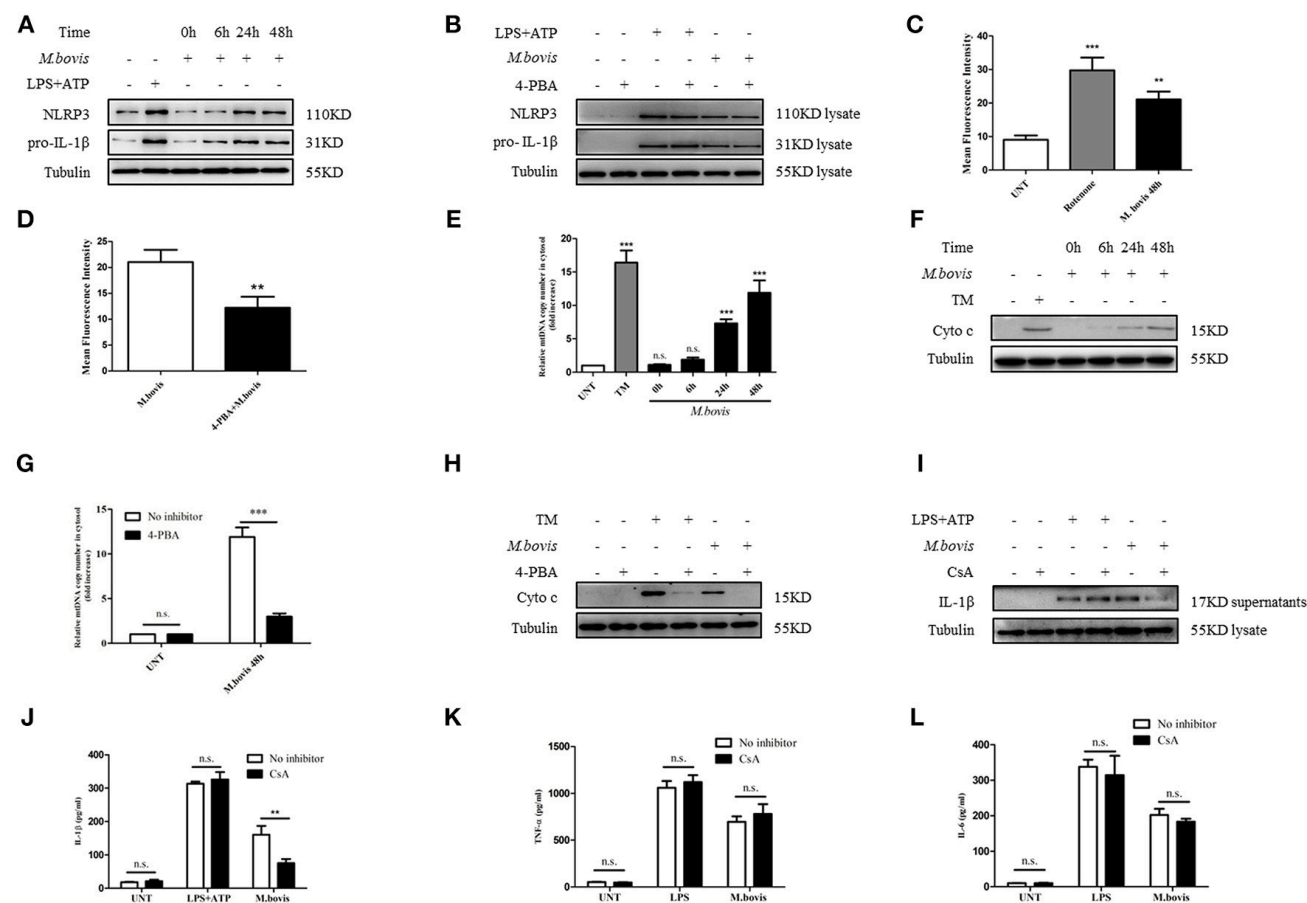
ERS-induced mitochondrial dysfunction is involved in inflammasome activation. **(A)** Immunoblot analysis at different time-points of NLRP3 and pro-IL-1β in lysates of BMDMs infected with *M. bovis* (MOI 10). **(B)** Immunoblot analysis of NLRP3 and pro-IL-1β in lysates from BMDMs infected for 24 h with *M. bovis* (MOI 10) in the presence or absence of 4-PBA. Control image is the same as Figure 1D. **(C)** ROS production measured at various time-points by flow cytometry in BMDMs infected with *M. bovis* (MOI 10). **(D)** ROS production was measured by flow cytometry in BMDMs infected for 48 h with *M. bovis* (MOI 10) in the presence or absence of 4-PBA. **(E)** qPCR analysis of mtDNA release into the cytosol during *M. bovis* (MOI 10) infection. **(F)** Immunoblot analysis at different time-points of cytochrome c in cytosolic extracts of BMDMs infected with *M. bovis* (MOI 10). **(G)** qPCR analysis of mtDNA release into the cytosol in BMDMs treated with or without 4-PBA for 1 h and then infected with *M. bovis* (MOI 10) for 48 h. **(H)** Immunoblot analysis of cytochrome c in cytosolic extracts from BMDMs infected with *M. bovis* (MOI 10) for 48 h in the absence or presence of 4-PBA. **(I)** IL-1β immunoblot analysis of supernatants from BMDMs infected for 24 h with *M. bovis* (MOI 10) in the presence or absence of CsA. **(J)** ELISA for IL-1β detection in supernatants from BMDMs treated with or without CsA for 1 h and then infected with *M. bovis* (MOI 10) for 24 h. Control image is the same to Figure 1E. ELISA for **(K)** TNF-α and **(L)** IL-6 quantification in supernatants from BMDMs treated with or without CsA for 1 h and then infected with *M. bovis* (MOI 10) for 24 h. Control image is the same to Figures 1H,I. LPS+ATP, positive control for inflammasome activation, 200 ng/ml, and 1 mM, respectively; LPS, positive control for TNF-α and IL-6 production, 200 ng/ml; 4-PBA, 4-phenyl butyric acid, ERS inhibitor, 5 mM; UNT, untreated; rotenone, positive control for ROS production, 40 μM; TM, tunicamycin, positive control for ERS-induced mitochondrial damage, 10 μg/mL; CsA, cyclosporine A, inhibitor of MPTP opening, 10 μM; siNLRP3, silencing RNA for NLRP3, 50 nM; siAIM2, silencing RNA for AIM2, 50 nM; siCon, non-targeting control siRNA, 50 nM. MOI, multiplicity of infection. For **(C,E)**, data are representative of at least three independent experiments. The results are shown as the mean ± SD. The asterisks indicate statistically significant differences compared with untreated cells (^**^P < 0.01, ^***^P < 0.001, n.s., not significant). *P*-values were analyzed by using one-way ANOVA followed by *post-hoc* Tukey's test. For **(D,G,J–L)**, data are representative of at least three independent experiments, each performed in triplicate for WB. The results are shown as mean ± SD. ^**^P < 0.01, ^***^P < 0.001, n.s., not significant. *P*-values were analyzed by using Student's *t*-test.

ERS might induce IL-1β production through the expression of NLRP3 and pro-IL-1β (25). We observed that NLRP3 and pro-IL-1β expression significantly increased during *M. bovis* infection (Figure 2A; Supplementary Figure 1A). We initially assessed whether *M. bovis*-induced the ERS-activated inflammasome through NLRP3 and pro-IL-1β expression through the evaluation of lysates from *M. bovis*-infected BMDMs in the presence or absence of 4-PBA. ERS did not contribute to *M. bovis*-triggered NLRP3 and pro-IL-1β expression (Figure 2B; Supplementary Figure 1B). ERS induces the release of mitochondria-derived damage-associated molecular patterns (mtDAMPs), which can activate the inflammasome (26–28). We observed that ERS induced ROS production in *M. bovis*-infected macrophages (Figures 2C,D; Supplementary Figures 1C,D), which is a typical phenotype of mitochondrial dysfunction; we first investigated the ultrastructural phenotypes of BMDMs after *M. bovis* infection. We found that *M. bovis* infection induced an abundance of swollen mitochondria with severely disrupted cristae in BMDMs, a phenomenon inhibited by 4-PBA (Supplementary Figure 1E). Subsequently, we infected BMDMs with *M. bovis* and measured cytochrome c and mtDNA release. *M. bovis*-infected BMDMs increased the mtDNA copy number and the level of cytochrome c into the cytosol (Figures 2E,F), whereas 4-PBA treatment blocked this process (Figures 2G,H). The release of mtDNA to the cytosol was reported to induce inflammasome activation (27). Here, we sought to investigate whether mtDNA released into the cytosol during *M. bovis* infection contributed to inflammasome activation. We treated BMDMs with or without cyclosporine A (CsA), an inhibitor of mitochondrial permeability transition pore opening, prior to *M. bovis* infection and measured IL-1β production. CsA treatment significantly decreased IL-1β production in *M. bovis*-infected macrophages (Figures 2I,J). Moreover, CsA treatment had no effect on the production of TNF-α and IL-6 (Figures 2K,L). The purity of the fractions was assessed by western blotting assay (Supplementary Figure 2A). These data suggested that *M. bovis* infection induced mitochondrial damage, which led to the activation of the inflammasome, as a result of the increase in ERS.

### NLRP3 and AIM2 Mediates ERS-Induced Mitochondrial Damage

**Figure 3:**
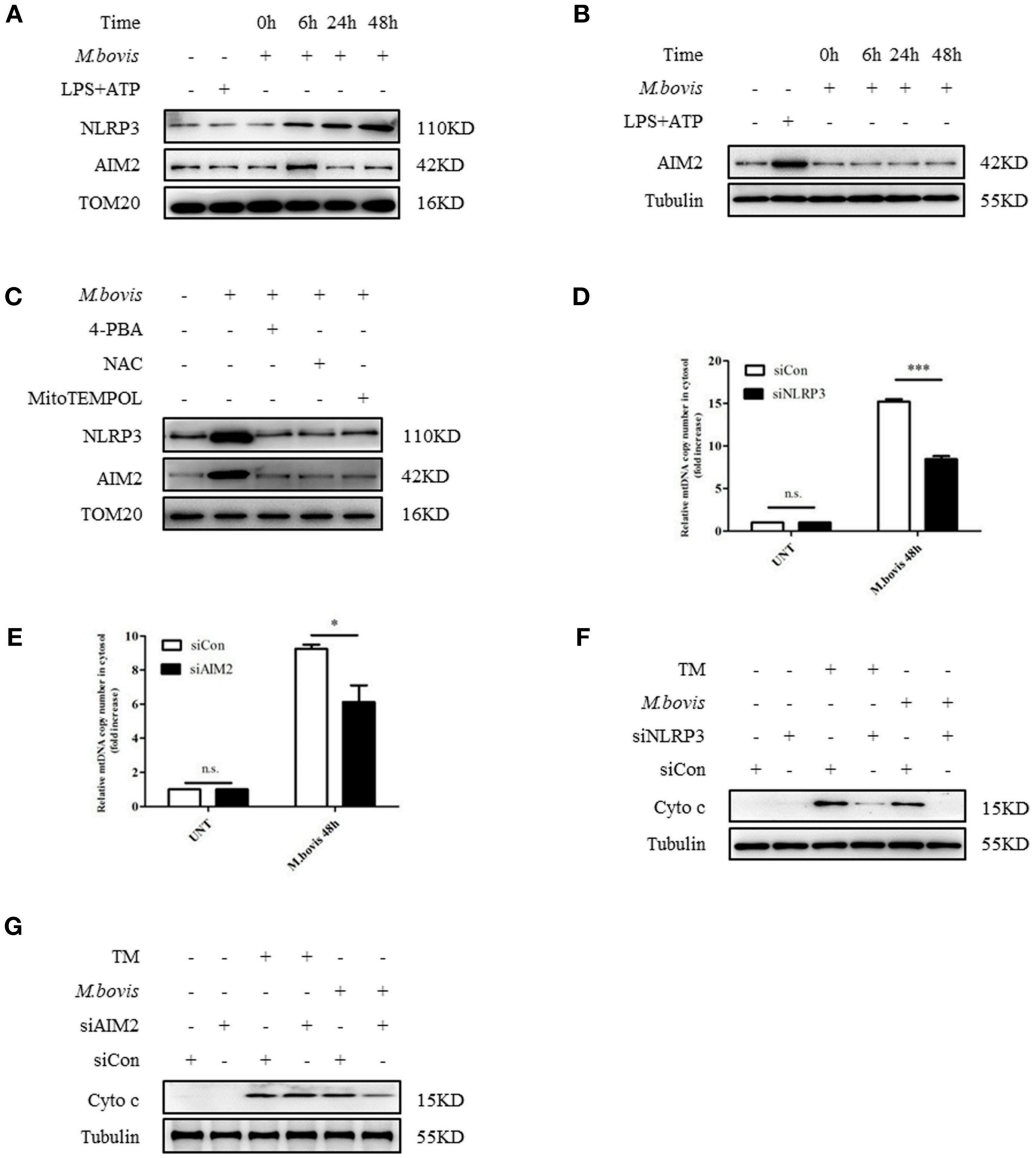
NLRP3 and AIM2 mediate ERS-induced mitochondrial damage. **(A)** Immunoblot analysis at different time points of NLRP3 and AIM2 at the mitochondrial fraction of BMDMs infected with *M. bovis* (MOI 10). **(B)** Immunoblot analysis at different time points of AIM2 in lysates of BMDMs infected with *M. bovis* (MOI 10). The control image is the same to Figure 2A. **(C)** Immunoblot analysis of NLRP3 and AIM2 at the mitochondrial fraction of BMDMs infected for 6 h with *M. bovis* (MOI 10) in the presence or absence of 4-PBA, NAC and Mito TEMPOL. **(D)** qPCR analysis of mtDNA release into the cytosol in *M. bovis*-infected BMDMs transfected with control non-targeting siRNA (siCon) or NLRP3-targeting siRNA (siNLRP3) and then infected for 48 h with *M. bovis* (MOI 10). **(E)** qPCR analysis of mtDNA release into the cytosol in *M. bovis*-infected BMDMs transfected with control non-targeting siRNA (siCon) or AIM2-targeting siRNA (siAIM2) and then infected for 48 h with *M. bovis* (MOI 10). **(F)** Immunoblot analysis of cytochrome c in cytosolic extracts from BMDMs transfected with control non-targeting siRNA or NLRP3 targeting siRNA and then infected for 48 h with *M. bovis* (MOI 10). **(G)** Immunoblot analysis of cytochrome c in cytosolic extracts from BMDMs transfected with control non-targeting siRNA (siCon) or AIM2 targeting siRNA (siAIM2) and then infected for 48 h with *M. bovis* (MOI 10). LPS+ATP, positive control for inflammasome activation, 200 ng/mL and 1 mM, respectively; 4-PBA, 4-phenyl butyric acid, ERS inhibitor, 5 mM; NAC, N-acety1-L-cysteine, the ROS scavenger, 5 mM; MitoTEMPOL, 4-hydroxy-2,2,6,6-tetramethylpiperidine-N-oxyl, mitochondria-targeted antioxidant agent, 500 μM; UNT, untreated; siNLRP3, silencing RNA for NLRP3, 50 nM; siAIM2, silencing RNA for AIM2, 50 nM; siCon, non-targeting control siRNA, 50 nM. MOI, multiplicity of infection. For **(D,E)**, the data are representative of at least three independent experiments, each measured in triplicate. The results are shown as the mean ± SD. ^*^P < 0.05, ^***^P < 0.001, n.s., not significant. *P*-values were analyzed by using Student's *t*-test.

Previous studies reported that in macrophages infected with *Mtb*, both NLRP3 and AIM2 regulated IL-1β production (29, 30). Moreover, the induction of the AIM2 inflammasome is required for IL-1β production in *M. bovis*-infected macrophages (21). Upon activation, NLRP3 and AIM2 translocate from the cytoplasm to the mitochondria and induce mitochondrial dysfunction (27, 28, 31). To determine whether NLRP3 and AIM2 contributed to the release of mitochondrial components in *M. bovis*-infected macrophages, we assessed whether NLRP3 and AIM2 were recruited to mitochondria. We observed that NLRP3 and AIM2 were recruited to the mitochondrial fraction at 6 h post infection in BMDMs, in the absence of increased expression (Figures 2A, 3A,B). The recruitment of NLRP3 and AIM2 to the mitochondrial fraction was significantly decreased in BMDMs pretreated with 4-PBA, NAC, the ROS scavenger, and 4-hydroxy-2,2,6,6-tetramethylpiperidine-N-oxyl (MitoTEMPOL), the mitochondria-targeted antioxidant agent (Figure 3C). Next, we investigated the effect of NLRP3 and AIM2 on the release of mitochondrial components in macrophages. We used small interfering RNA to suppress the expression of NLRP3 and AIM2 in BMDMs (Supplementary Figure 2B). The release of mtDNA and cytochrome c induced by *M. bovis* infection was blocked by NLRP3 and AIM2 silencing (Figures 3D–G). These findings suggested that during *M. bovis* infection, ERS-induced mitochondrial damage was mediated by NLRP3 and AIM2.

### NLRP3 Induces Mitochondrial Damage Via Caspase-8

**Figure 4:**
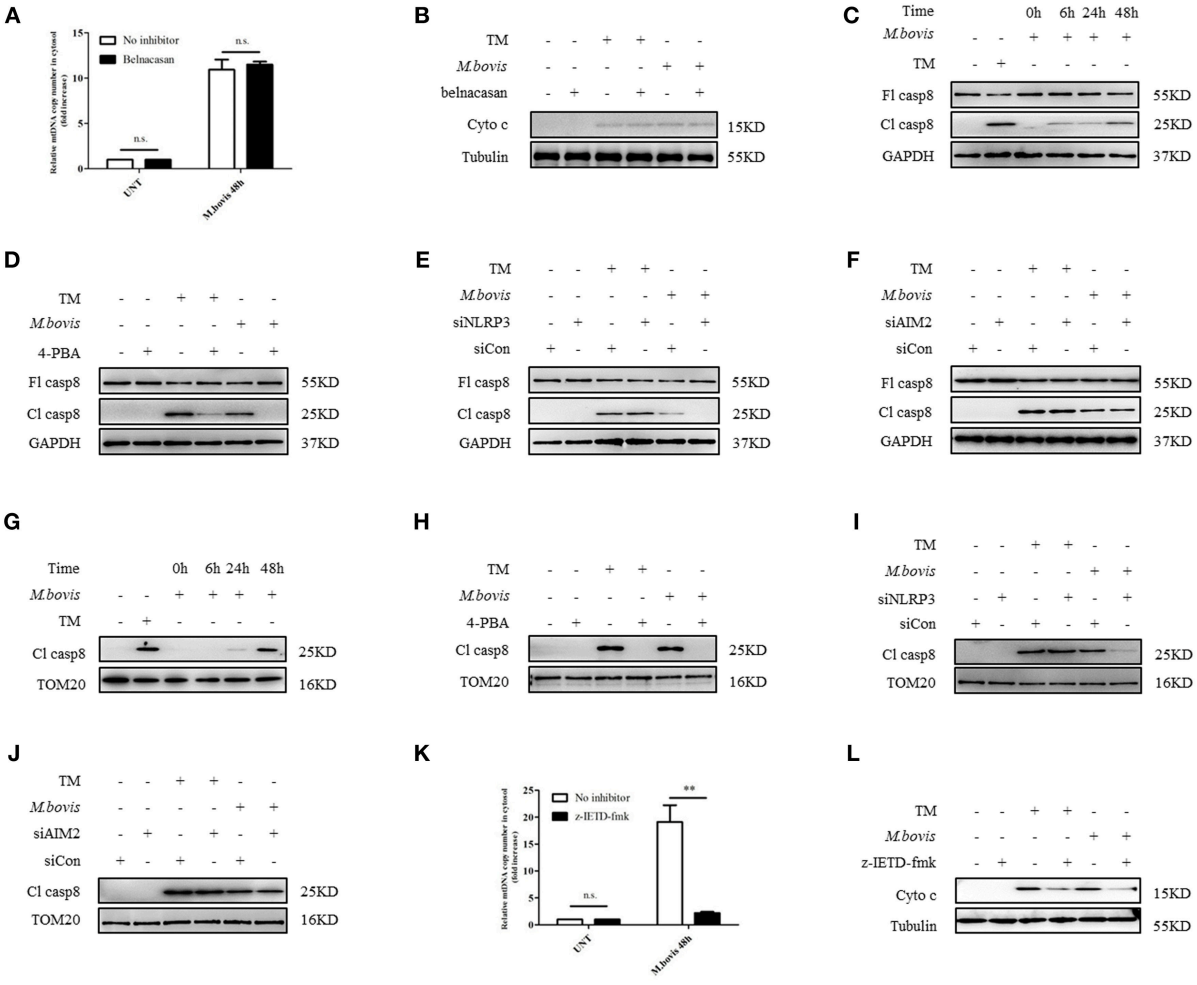
NLRP3 and caspase-8 are required for ERS-induced mitochondrial damage. **(A)** qPCR analysis of mtDNA release into the cytosol in BMDMs treated with no inhibitor and belnacasan for 1 h and then infected with *M. bovis* (MOI 10) for 48 h. **(B)** Immunoblot analysis of cytochrome c in the cytosolic extracts from BMDMs treated with no inhibitor or belnacasan for 1 h and then infected with *M. bovis* (MOI 10) for 48 h. **(C)** Immunoblot analysis at different time points of caspase-8 in lysates of BMDMs infected with *M. bovis* (MOI 10). **(D)** Immunoblot analysis of caspase-8 in lysates of BMDMs treated with no inhibitor or 4-PBA for 1 h and then infected with *M. bovis* (MOI 10) for 48 h. **(E)** Immunoblot analysis of caspase-8 in lysates of BMDMs transfected with control non-targeting siRNA (siCon) or NLRP3 targeting siRNA (siNLRP3) and then infected for 48 h with *M. bovis* (MOI 10). **(F)** Immunoblot analysis of caspase-8 in lysates of BMDMs transfected with siCon or siAIM2 and then infected for 48 h with *M. bovis* (MOI 10). **(G)** Immunoblot analysis at different time points of cleaved caspase-8 in the mitochondrial fraction of BMDMs infected with *M. bovis* (MOI 10). **(H)** Immunoblot analysis of caspase-8 in the mitochondrial fraction of BMDMs infected with *M. bovis* (MOI 10) for 48 h in the presence or absence of 4-PBA. **(I)** Immunoblot analysis of caspase-8 in the mitochondrial fraction of BMDMs transfected with control non-targeting siRNA (siCon) or NLRP3 (siNLRP3) targeting siRNA and then infected for 48 h with *M. bovis* (MOI 10). **(J)** Immunoblot analysis of caspase-8 in the mitochondrial fraction of BMDMs transfected with control non-targeting siRNA (siCon) or AIM2 targeting siRNA (siAIM2) and then infected for 48 h with *M. bovis* (MOI 10). **(K)** qPCR analysis of mtDNA release into the cytosol in BMDMs treated with no inhibitor or z-IETD-fmk for 1 h and then infected with *M. bovis* (MOI 10) for 48 h. **(L)** Immunoblot analysis of cytochrome c in cytosolic extracts from BMDMs infected with *M. bovis* (MOI 10) in the absence or presence of z-IETD-fmk. UNT, untreated; belnacasan, inhibitor of caspase-1, 20 μM; TM, tunicamycin, positive control for ERS-induced caspase-8 cleavage, 10 μg/mL; 4-PBA, 4-phenyl butyric acid, ERS inhibitor, 5 mM; z-IETD-fmk, caspase-8 inhibitor, 50 μM; Fl casp8, full-length caspase-8; Cl casp8, cleaved caspase-8; siNLRP3, silencing RNA for NLRP3, 50 nM; siAIM2, silencing RNA for AIM2, 50 nM; siCon, non-targeting control siRNA, 50 nM. MOI, multiplicity of infection. The data are representative of at least three independent experiments, each measured in triplicate. The results are shown as the mean ± SD. ^**^P < 0.01, n.s., not significant. *P*-values were analyzed by using Student's *t*-test.

It has been shown that in *M. bovis-*infected macrophages, caspase-1 is required for IL-1β production (21). Moreover, caspase-1 can drive mitochondrial dysfunction when stimulated by ATP or transfected with DNA (31). Therefore, we assessed whether caspase-1 is required by *M. bovis*-induced mitochondrial dysfunction. We cultured *M. bovis-*infected BMDMs in the presence and absence or belnacasan, a caspase-1 inhibitor, and measured the release of mitochondrial components; no significant reduction in the release of mtDNA (Figure 4A) and cytochrome c in the cytosolic fraction of macrophages was found (Figure 4B). It is suggested that TNF-α treatment induced caspase-8 cleavage through NLRP3 inflammasome activation, which resulted in mitochondrial dysfunction and the subsequent release of cytochrome c (32). In addition, it is reported that ERS or *M. bovis* infection induced the activation of caspase-8 (21, 33). We reasoned that under ERS, NLRP3, and AIM2 might be involved in the activation of caspase-8 and the subsequent release of mitochondrial content. We assessed whether caspase-8 was activated during *M. bovis* infection through the measurement of cleaved active caspase-8. A relative increase in the expression of caspase-8 cleavage was observed in *M. bovis*-infected BMDMs at 6 h post-infection (Figure 4C). We then probed the lysates from no inhibitor-treated and 4-PBA-treated infected BMDMs to investigate the role of ERS in caspase-8 activation. We found that ERS induced caspase-8 cleavage during *M. bovis* infection (Figure 4D). To determine whether NLRP3 and AIM2 acted upstream of caspase-8, control, NLRP3-silenced, and AIM2-silenced BMDMs were infected with *M. bovis* and probed for cleaved caspase-8. Caspase-8 cleavage significantly decreased in infected NLRP3-silenced BMDMs, although the silencing of AIM2 had no clear effect on the cleavage of caspase-8 compared with the control (Figures 4E,F). We then investigated whether NLRP3 was required for ERS initiation through the measurement of the expression of ERS markers. No significant decrease in Bip or phosphorylated IRE1α was observed in infected NLRP3-silenced BMDMs (Supplementary Figures 2C,D). Subsequently, we investigated the mitochondrial recruitment of active caspase-8 and its role in mitochondrial dysfunction. We detected that cleaved caspase-8 was recruited to the mitochondrial fraction (Figure 4G), but that this recruitment was abolished by 4-PBA treatment or in NLRP3-silenced macrophages (Figures 4H,I), but not in AIM2-silenced macrophages (Figure 4J). BMDMs treated with z-IETD-fmk, a caspase-8 inhibitor, released less mitochondrial content into the cytosol (Figures 4K,L). These data suggested that during *M. bovis* infection, NLRP3, but not AIM2, mediated ERS-induced mitochondrial dysfunction through a caspase-8 dependent pathway.

### NLRP3 and Caspase-8 Drive Mitochondrial Dysfunction Through the Pore-Activating Factor Bid

**Figure 5:**
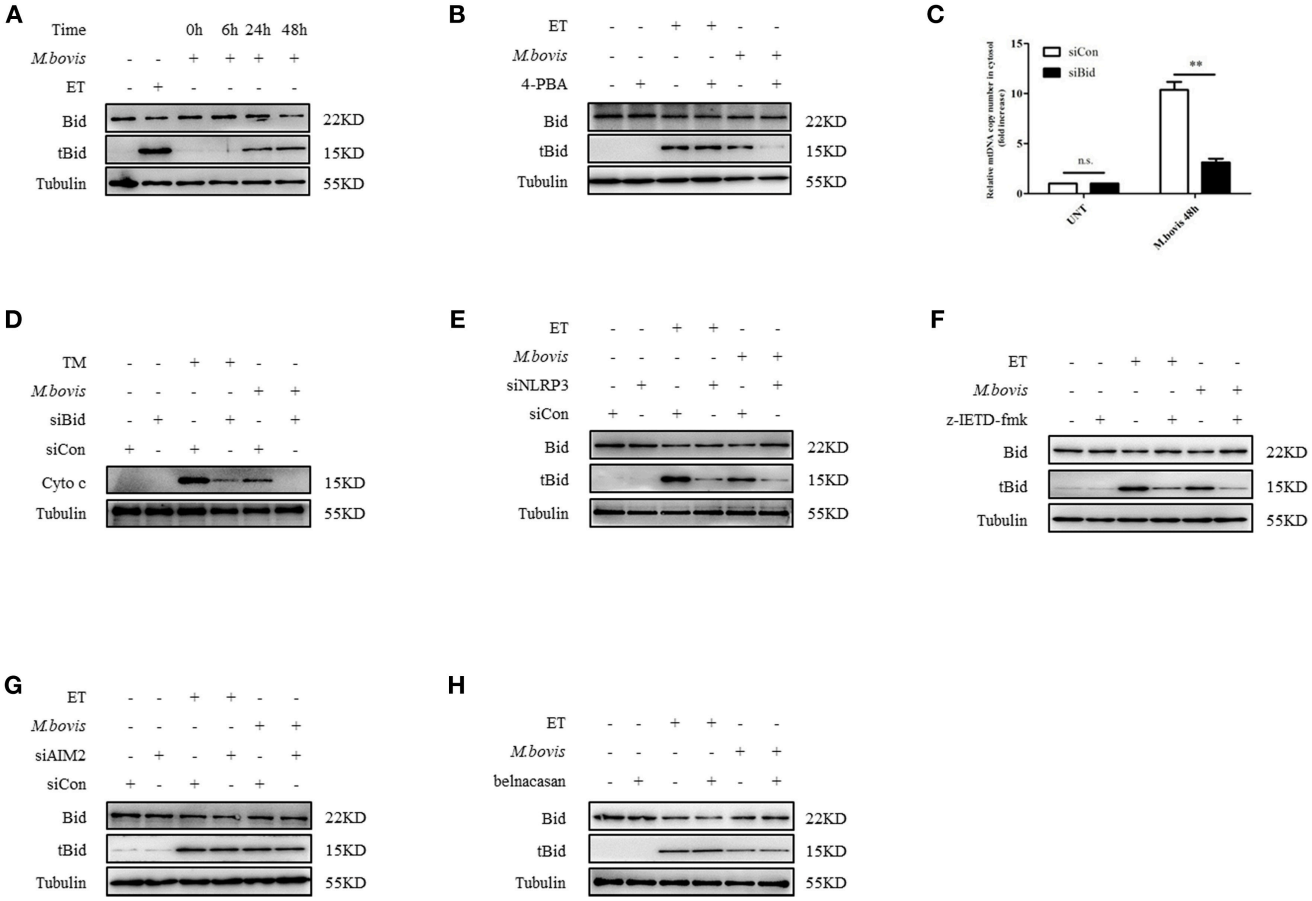
NLRP3 and caspase-8 drive mitochondrial dysfunction through the pore-activating factor, Bid. **(A)** Immunoblot analysis at different time points of Bid in lysates of BMDMs infected with *M. bovis* (MOI 10). **(B)** Immunoblot analysis of Bid in lysates of BMDMs infected with *M. bovis* (MOI 10) in the presence or absence of 4-PBA. **(C)** qPCR analysis of mtDNA in cytosolic extracts from BMDMs transfected with control non-targeting siRNA (siCon) or Bid targeting siRNA (siBid) followed by *M. bovis* (MOI 10) infection for 48 h. **(D)** Immunoblot analysis of cytochrome c in cytosolic extracts from BMDMs transfected with control non-targeting siRNA (siCon) or Bid targeting siRNA (siBid) followed by *M. bovis* (MOI 10) infection for 48 h. **(E)** Immunoblot analysis of Bid in lysates of BMDMs transfected with control non-targeting siRNA (siCon) or NLRP3 targeting siRNA (siNLRP3) and then infected with *M. bovis* (MOI 10) for 48 h. **(F)** Immunoblot analysis of Bid in lysates of BMDMs treated with or without z-IETD-fmk for 1 h and then infected with *M. bovis* (MOI 10) for 48 h. **(G)** Immunoblot analysis of Bid in lysates of BMDMs transfected with control non-targeting siRNA (siCon) or AIM2 targeting siRNA (siAIM2) and then infected with *M. bovis* (MOI 10) for 48 h. **(H)** Immunoblot analysis of Bid in lysates of BMDMs treated with or without belnacasan for 1 h and then infected for 48 h with *M. bovis* (MOI 10). ET, etoposide, positive control for Bid truncation, 25 μM; 4-PBA, 4-phenyl butyric acid, ERS inhibitor, 5 mM; UNT, untreated; TM, tunicamycin, positive control for ERS induced mitochondrial damage, 10 μg/ml, z-IETD-fmk, caspase-8 inhibitor, 50 μM. Belnacasan, inhibitor of caspase-1, 20 μM; siNLRP3, silencing RNA for NLRP3, 50 nM; siAIM2, silencing RNA for AIM2, 50 nM; siBid, silencing RNA for Bid, 50 nM; siCon, non-targeting control siRNA, 50 nM. MOI, multiplicity of infection. Data are representative of at least three independent experiments. The results are shown as the mean ± SD. ^**^P < 0.01, n.s., not significant. *P*-values were analyzed by using Student's *t*-test.

We aimed to ascertain the pathway through which NLRP3 and caspase-8 drove mitochondrial dysfunction. NLRP3 and caspase-8 could induce the cleavage and activation of Bid, which induces mitochondrial damage by facilitating pore formation of Bax (32, 34). We hypothesized that under ERS, NLRP3, and caspase-8 might induce Bid truncation, which leads to mitochondrial membrane permeabilization. We first assessed whether Bid was cleaved during *M. bovis* infection. We observed increased truncation of Bid in BMDMs at 24 h post-*M. bovis* infection (Figure 5A). To determine the role of Bid in ERS-derived mitochondrial dysfunction, we probed lysates from infected BMDMs in the presence or absence of 4-PBA treatment for tBid; with etoposide (ET) used as a positive control. 4-PBA treatment decreased tBid in *M. bovis*-infected, but not in ET-treated BMDMs (Figure 5B). Infected Bid-silenced BMDMs (Supplementary Figure 2E) released less mtDNA (Figure 5C) and cytochrome c (Figure 5D) into the cytosol than controls, indicating the importance of Bid in ERS-induced mitochondrial damage. To determine the role of NLRP3 and caspase-8 in this process, tBid was assessed in NLRP3-silenced and z-IETD-fmk-treated infected BMDMs. Bid truncation was significantly decreased in these macrophages (Figures 5E,F), but not in AIM2-silenced and belnacasan-treated cells (Figures 5G,H). These data suggested that during *M. bovis* infection, NLRP3 and caspase-8 regulated mitochondrial damage via Bid. Treatment with inhibitor or siRNA exerted no effect on macrophage viability and the bacterial uptake of macrophages (Supplementary Figures 2F,G).

### ERS Mediates *M. bovis*-induced Inflammasome Activation *in vivo*

**Figure 6:**
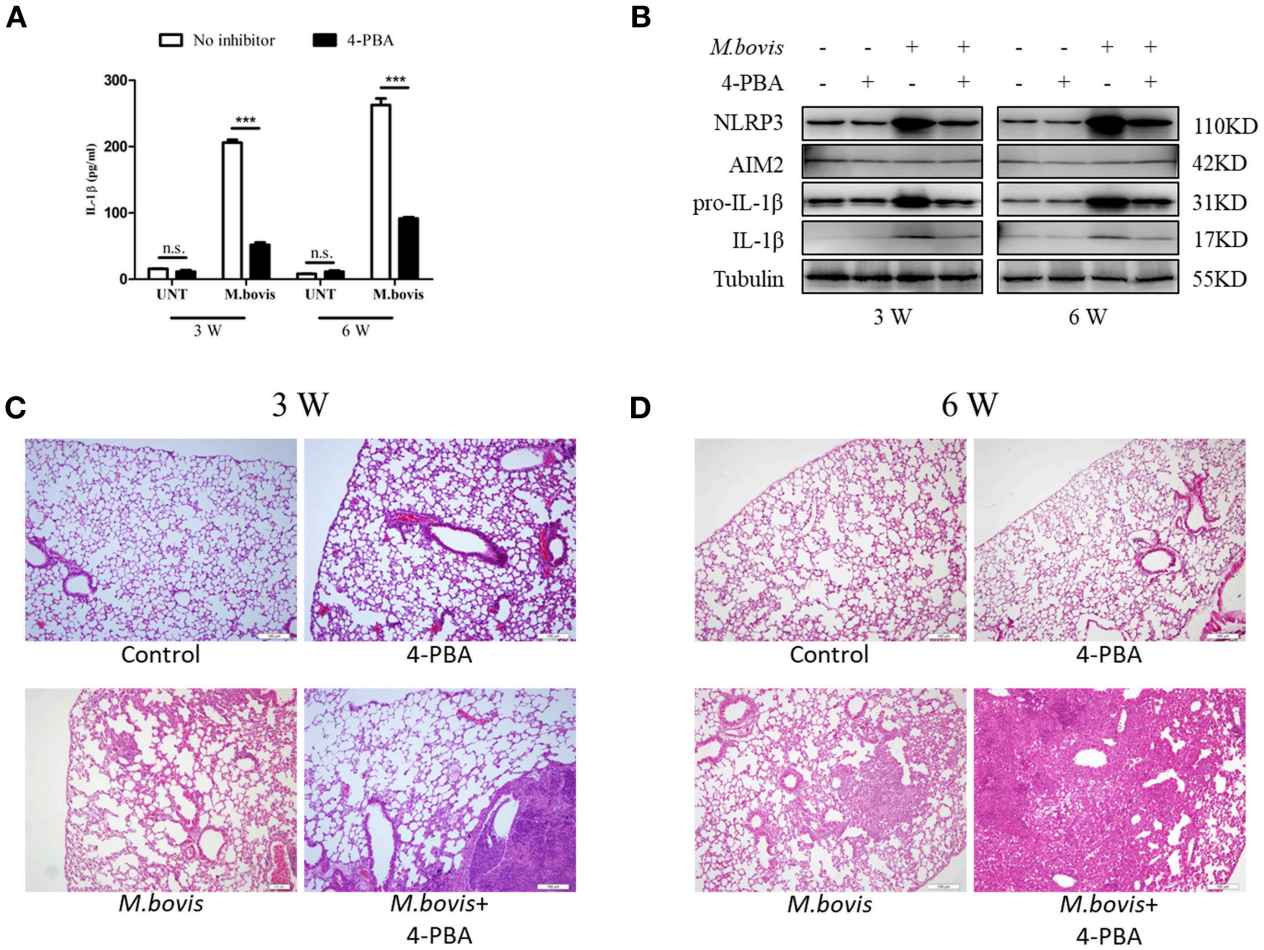
ERS mediates *M. bovis*-induced inflammasome activation *in vivo*. **(A)** ELISA detection of IL-1β in serum samples from C57BL/6 mice treated with or without 4-PBA (18.6 mg/mouse/day) and infected with *M. bovis* (CFU 200) (*n* = 7). **(B)** Immunoblot analysis of NLRP3, AIM2, pro-IL-1β, and IL-1β in the lung tissues of *M. bovis*-infected mice in the presence or absence of 4-PBA. **(C,D)** Pathological lesions (H&E staining) in the lung of mice infected with *M. bovis* for 3 weeks **(C)** or 6 weeks **(D)** in the presence or absence of 4-PBA (18.6 mg/mouse/day) (*n* = 3). 4-PBA, 4-phenyl butyric acid, ERS inhibitor, 18.6 mg/mouse/day; CFU, colony forming units (*n* = 3). The results are shown as the mean ± SD. ^***^P < 0.001, n.s., not significant. *P*-values were analyzed by using Student's *t*-test **(C,D)**. Scale bar = 100 μm.

*Mtb* infection induced ERS in the lungs of mice (35). Our findings suggested that ERS triggered inflammasome activation *in vitro* during *M. bovis* infection. To determine whether ERS contributed to the *M. bovis*-induced inflammasome activation *in vivo*, we infected C57BL/6 mice with *M. bovis* in the presence or absence of 4-PBA. Animals were sacrificed at 3 weeks and 6 weeks post-infection, and serum and lung tissues were collected aseptically. *M. bovis* infection induced a significant increase in the production of IL-1β in the serum and lungs, a phenotype which was reversed by 4-PBA treatment (Figures 6A,B). In addition, *M. bovis* infection induced a significant TNF-α increase in the serum, but 4-PBA treatment did not affect the serum level of TNF-α (Supplementary Figure 3A). Intriguingly, we observed a decrease in NLRP3 and pro-IL-1β expression in the lungs of *M. bovis*-infected mice treated with 4-PBA (Figure 6B). Subsequently, we investigated the role of ERS in *M. bovis*-induced pathological injury. The administration of 4-PBA aggravated lung lesions, leading to more extensive nidus, massive inflammatory cell infiltration (Figures 6C,D; Supplementary Figure 3B) and more severe bacterial burden (Supplementary Figure 3C), which indicated the protective role of ERS during infection. Collectively, these data showed that in *M. bovis*-infected mice, ERS has a critical role in the reduction of disease severity via IL-1β production.

## Discussion

Additional ERS is relevant to human health as the crosstalk between ERS and inflammation has been increasingly implicated in various acute and chronic diseases, including infection, neurodegenerative disorders, and type 2 diabetes (36). The results of our study have provided mechanistic insights into the relative role of ERS, mitochondrial dysfunction, and inflammasome activation in the regulation of IL-1β during *M. bovis* infection. The principal findings of this study were: (1) ERS induced by *M. bovis* infection was involved in IL-1β production; (2) ERS damaged mitochondria through an inflammasome-dependent mechanism during *M. bovis* infection; and (3) damaged mitochondria drive inflammasome activation and IL-1β production through the release of DAMPs, mtDNA, and cytochrome c. To the best of our knowledge, this is the first report to investigate the role of *M. bovis* infection-induced ERS in mitochondrial damage and inflammasome activation that lead to IL-1β production.

Previous studies have demonstrated that Ca^2+^ signaling may be a common mechanism by which diverse stressors, including K^+^ efflux, ROS, and lysosome destabilization, activate inflammasomes (37). Lee et al. added Ca^2+^ to lysates of LPS-stimulated macrophages and observed that Ca^2+^ binding to the NLRP3 inflammasome complex directly facilitated the co-immunoprecipitation of ASC and NLRP3. The study of Lee et al. showed that Ca^2+^ triggers inflammasome activation independent of mitochondrial function (38). In the context of our study, inflammasome activation by *M. bovis* infection-induced ERS was essentially dependent on the release of mitochondrial content. To reconcile the contradiction between our data and previously reported results, we speculated that the concentration of cytosolic Ca^2+^ might be critical role in the dependence of inflammasome activation on mitochondrial dysfunction. Stress that induced significant elevation of cytosolic Ca^2+^ concentration might induce inflammasome activation without mitochondrial damage. Stress that results in a weak induction of cytosolic Ca^2+^ concentration might be more dependent on mitochondrial dysfunction.

ROS may induce the activation of NLRP3 inflammasome as well as recruiter molecules, which results in the translocation of NLRP3 to the mitochondria, and leads the NLRP3 inflammasome to cleave Bid and trigger mitochondrial damage. Notably, our studies have shown that in *M. bovis*-infected macrophages, AIM2 inflammasome activation did not require ROS (21). However, the recruitment of AIM2 inflammasome to mitochondria was essentially dependent on ROS. Upon recruitment, NLRP3 binds the exposed mitochondrial phospholipid, cardiolipin (26, 39), or the mitochondrial antiviral signaling protein, MAVS, which serve as “scaffolding” for complex assembly (40). Hence, it will be of interest to determine whether cardiolipin and MAVS were more broadly involved in binding to other inflammasomes, including AIM2.

The NLRP3 and AIM2 inflammasomes recruit and cleave pro-caspase-1; subsequently, active caspase-1 leads rapidly to lytic death, referred to as pyroptosis. Our study indicated that *M. bovis* infection induced caspase-1-dependent pyroptosis (21), but the mechanism through which caspase-1 triggers cell death is still unknown. Caspase-1 is reported to drive mitochondrial dysfunction, leading to pyroptosis (31). However, a recent report indicated that caspase-1 was not involved in mitochondrial dysfunction in response to ERS (41), which agreed with our data in BMDMs, suggesting that an alternative caspase, other than caspase-1, is the key proteinase that induces mitochondrial damage. To reconcile these contradictory data, we speculated that caspase-1 and caspase-8 might catalyze common substrates (i.e., Bid and pro-IL-1β), and that the catalytic efficiency might differ for different substrates. In contrast with caspase-1, caspase-8 strongly catalyzes Bid cleavage and weakly catalyzes IL-1β production. Stimuli that activate caspase-8 might allow the cleavage of Bid, followed by mitochondrial dysfunction, without a requirement for caspase-1. Stimuli that cannot trigger caspase-8 activation might be dependent on caspase-1 for Bid truncation. Consistent with our speculation, in conditions in which caspase-1 is not activated, caspase-8 is utilized as the major IL-1β-converting protease (33, 42, 43).

Bacterial infection induces complex stresses on host cells that are not completely explained for most microorganisms; however, they are known to incorporate oxidative stress, organelle perturbations, K^+^ efflux, and nutrient deprivation. Early studies reported that microorganisms induce ERS (19, 20, 33, 44), whereas ERS and NLRP3 are important regulators of the protective immune responses against microbial infection (19, 20). These observations indicated that the modulation of ERS may offer a promising strategy for the resistance to infection through the regulation of inflammatory responses. Importantly, as ERS is associated with sterile inflammatory diseases, such as obesity, type 2 diabetes, Crohn's disease, and cancer (45), and the production of various cytokines, it may be important in the investigation of whether the NLRP3-caspase-8 axis and mitochondrial dysfunction are more intricately engaged with these mechanisms.

NLRP3 has emerged as a mediator of mitochondrial damage and ERS. The role of ERS in the NLRP3-caspase-8-induced mitochondrial dysfunction has still not been elucidated. NLRP3 is recruited to mitochondria, suggesting that the recognition of mitochondrial lipids or proteins, which may serve as DAMPs, by NLRP3, together with the subsequent interaction between these molecules, might be a possible mechanism that leads to NLRP3 activation and caspase-8 cleavage. It will be of interest to determine the role of ERS in mitochondrial lipid/protein synthesis and translocation during *M. bovis* infection. We found that AIM2 was activated and recruited to mitochondria during *M. bovis* infection. Unexpectedly, AIM2 was not critically important for caspase-8 cleavage or Bid truncation. The most probable explanation is that cells exploit numerous parallel or redundant pathways, including but not limited to the caspase-8-Bid pathway, to trigger mitochondrial dysfunction. z-IETD-fmk is a selective and cell-permeable caspase-8 inhibitor. Unexpectedly, z-IETD-fmk partially inhibited the cleavage of caspase-3 (46). We speculated that the inhibitory effect of z-IETD-fmk on cleavage of caspase-3 was not a direct effect, but was mediated through the inhibition of caspase-8 cleavage. The inhibition of caspase-8 activation induced a decrease in cytochrome c release, a process that is responsible for the inhibition of the activation of apoptotic caspases. Yue et al. (47) showed that caspase-3 activation and Bid cleavage formed a positive feedback loop that is engaged by hyperosmotic shock to induce apoptosis in Xenopus oocytes. The exploration of the existence of such a caspase-3/Bid loop in *M. bovis*-infected macrophages would be an intriguing research topic.

We observed that 4-PBA treatment aggravated *M. bovis*-induced lung lesions, which indicated the protective role of ERS during infection. In addition, our results suggested that ERS enhanced the production of IL-1β. An emerging body of evidence suggests that IL-1β plays a key role in the control of mycobacterium infection (2–7), it is reasonable to speculate that ERS boosts immunologic defense through IL-1β secretion after *M. bovis* infection. The level of IL-1β secretion into the extracellular matrix is dependent on pro-IL-1β expression and inflammasome activation (8–10). We observed that 4-PBA exerted no influence on the expression of pro-IL-1β in *M. bovis*-infected BMDMs, which suggested that ERS-induced IL-1β production was not dependent on the expression of the precursor, but the activation of the inflammasome. However, in *M. bovis*-infected lung tissue, ERS inhibitor significantly decreased pro-IL-1β expression, which suggested that ERS increased the level of mature IL-1β through precursor generation and the possible activation of the inflammasome. We speculated that these contradictory results might result from the different mechanisms engaged by different cell types. IL-1β may be derived from several cell types such asmacrophages, epithelial cells, and neutrophils) and each cell type may have a partly-overlapping, yet unique, mechanism that mediated ERS and IL-1β production. The induction of ERS in macrophages might trigger IL-1β production through inflammasome activation, but not pro-IL-1β expression, whereas the induction of ERS in other cells might boost mature IL-1β through precursor augment and/or inflammasome assembly.

Moreover, the effect of 4-PBA on bacillary uptake other than the effect on IL-1β production is worthy of consideration. Although the treatment of 4-PBA did not influence the phagocytosis of BMDMs, other types of phagocytic cells may engage different phagocytosis mechanisms, which require 4-PBA for bacillary uptake. ERS and IL-1β secretion was inhibited by 4-PBA administration; in addition, bacillary uptake and pathological changes also increased in the infected lung tissues. Moreover, several important questions remain unanswered; for example, the nature of the particular mechanism mediating ERS and IL-1β secretion from other cell types *in vivo*; the involvement of ERS in the regulation of bacillary uptake in infected tissues; the role of ERS in the induction of potential inflammatory cytokines (i.e., IL-6, TNF-α, and IFN-β); and how these cytokines interact to regulate IL-β production and phagocytosis? The development of drug-like ERS inducers in the future may allow us to explore these questions and such drugs may be useful for in anti-tuberculosis therapy.

In conclusion, our results suggested that ERS-induced NLRP3-caspase-8 activation contributed to mitochondrial dysfunction, which in turn triggered NLRP3-caspase-1 activation and IL-1β secretion. These findings contribute to the understanding of the innate immune mechanisms during mycobacterial infection and lay the groundwork for the investigation of ERS regulation as a therapeutic strategy for infectious and sterile inflammatory diseases.

## Ethics Statement

All protocols and procedures were performed according to the Chinese Regulations of Laboratory Animals—The Guidelines for the Care of Laboratory Animals (Ministry of Science and Technology of People's Republic of China) and Laboratory Animal Requirements of Environment and Housing Facilities (GB 14925–2010, National Laboratory Animal Standardization Technical Committee). The license number associated with their research protocol was 20110611–01 and the animal study proposal was approved by The Laboratory Animal Ethical Committee of China Agricultural University.

## Author Contributions

YL performed the experiments and wrote the manuscript. TH, YC, and CL contributed to experiments design. JW, JY, HC, and YS helped in cell culture and animal infection. TH, NS, and MH assisted in the English grammar check. XZ and DZ guided the performance of experiments and reviewed the manuscript critically before submission.

### Conflict of Interest Statement

The authors declare that the research was conducted in the absence of any commercial or financial relationships that could be construed as a potential conflict of interest.

## Supplementary Material

Supplementary Figure 1 ERS-induced mitochondrial dysfunction is involved in inflammasome activation. In support of Figure 2; **(A)** qPCR analysis of *Il1b* and *Nlrp3* transcript in BMDMs infected with *M. bovis* (MOI 10). **(B)** qPCR analysis of *Il1b* and *Nlrp3* transcript in BMDMs infected for 24 h with *M. bovis* (MOI 10) in the presence or absence of 4-PBA. **(C)** ROS production was measured at different time points by using flow cytometry in BMDMs infected with *M. bovis* (MOI 10). **(D)** ROS production was measured by flow cytometry in BMDMs infected for 48 h with *M. bovis* (MOI 10) in the presence or absence of 4-PBA. **(E)** TEM analysis of BMDMs infected with *M. bovis* (MOI 10) for 48 h in the presence or absence of 4-PBA. Arrows indicate mitochondria before and after infection. The data are representative of at least three independent experiments, each performed in triplicate. LPS+ATP, positive control for inflammasome activation, 200 ng/mL and 1 mM, respectively; 4-PBA, 4-phenyl butyric acid, ERS inhibitor, 5 mM; UNT, untreated; MOI, multiplicity of infection.

Supplementary Figure 2 **(A)** Immunoblot analysis of tubulin, β-actin (a cytosolic marker), TOM20, and VDAC (a mitochondrial marker) in whole cell lysate (WCL), the cytosolic fraction of cells (Cyto), and the mitochondrial fraction (Mito). **(B)** Immunoblot analysis of the expression NLRP3 and AIM2 in BMDMs transfected with control non-targeting siRNA (siCon), NLRP3-targeting siRNA (siNLRP3), or AIM2-targeting siRNA (siAIM2). **(C)** Immunoblot analysis of Bip in BMDMs transfected with control non-targeting siRNA or NLRP3 targeting siRNA and then infected for 24 h with *M. bovis* (MOI 10). **(D)** Immunoblot analysis of IRE1α in BMDMs transfected with siCon or siNLRP3 and then infected for 6 h with *M. bovis* (MOI 10). **(E)** Immunoblot analysis of the expression of Bid in BMDMs transfected with siCon or Bid -targeting siRNA (siBid). **(F)** Cell viability of BMDMs in the presence or absence of various inhibitors or siRNA. Inhibitors were added to cells 1 h prior to *M. bovis* infection (MOI 10). siRNA transfection medium was added to cells 48 h prior to *M. bovis* infection (MOI 10) and replaced with fresh medium 24 h prior to infection. After infection for 2 h, the inoculum was removed. The cells were washed with PBS and cultured at 37°C in an atmosphere of 5% CO_2_. At the indicated time points, the cell viability was measured. **(G)** Cell phagocytic capacity of BMDMs in the presence or absence of various inhibitors or siRNA. Inhibitors were added to cells 1 h prior to *M. bovis* infection (MOI 10). siRNA transfection medium was added to cells 48 h prior to *M. bovis* infection (MOI 10) and replaced with fresh medium 24 h prior to infection. After 2 h of infection, the inoculum was removed. Cells were washed with PBS and then lysed to enumerate intracellular CFU. UNT, untreated; 4-PBA, 4-phenyl butyric acid, ERS inhibitor, 5 mM; NAC, N-acety1-L-cysteine, the ROS scavenger, 5 mM; MitoTEMPOL, 4-hydroxy-2,2,6,6-tetramethylpiperidine-N-oxyl, mitochondria-targeted antioxidant agent, 500 μM; CsA, cyclosporine A, inhibitor of MPTP opening, 10 μM; z-IETD-fmk, caspase-8 inhibitor, 50 μM; Belnacasan, inhibitor of caspase-1, 20 μM; siNLRP3, siAIM2m and siBid, silencing RNA for NLRP3, AIM2m, and Bid, respectively, 50 nM; siCon, control non-targeting siRNA; MOI, multiplicity of infection. For **(A,F,G)**, the data are representative of at least three independent experiments, each performed in triplicate. The results are shown as the mean ± SD; n.s., not significant. *P*-values were analyzed by using one-way ANOVA followed by *post-hoc* Tukey's test. For **(B)**, the data are representative of at least three independent experiments, each measured in triplicate. The results are shown as the mean ± SD. *P*-values were analyzed by using Student's *t*-test.

Supplementary Figure 3 ERS mediates *M. bovis*-induced inflammasome activation *in vivo*. Related to Figure 6. **(A)** ELISA analysis of serum TNF-α and IL-6 in mice treated with or without 4-PBA (18.6 mg/mouse/day) and infected with *M. bovis* (CFU 200) (*n* = 3). **(B)** Clinical scores of mice infected with *M. bovis* (CFU 200) for 3 weeks or 6 weeks in the presence or absence of 4-PBA (18.6 mg/mouse/day). **(C)** Bacterial burden (acid-fast staining) in the lung of mice infected with *M. bovis* (CFU 200) for 3 weeks or 6 weeks in the presence or absence of 4-PBA (18.6 mg/mouse/day). 4-PBA, 4-phenyl butyric acid, ERS inhibitor, 18.6 mg/mouse/day; CFU, colony forming units (*n* = 3). The data shown are the mean ± SD. ^***^*P* < 0.001, n.s., not significant. *P*-values were analyzed by using Student's *t*-test. **(C)** Scale bar = 10 μm.
