# Supplementary figures and images for: Endoplasmic Reticulum Stress Induces Macrophages to Produce IL-1β During Mycobacterium bovis Infection via a Positive Feedback Loop Between Mitochondrial Damage and Inflammasome Activation
Source: Front Immunol. 2019 Feb 21;10:268. doi: 10.3389/fimmu.2019.00268 (PMC6394253; doi:10.3389/fimmu.2019.00268)

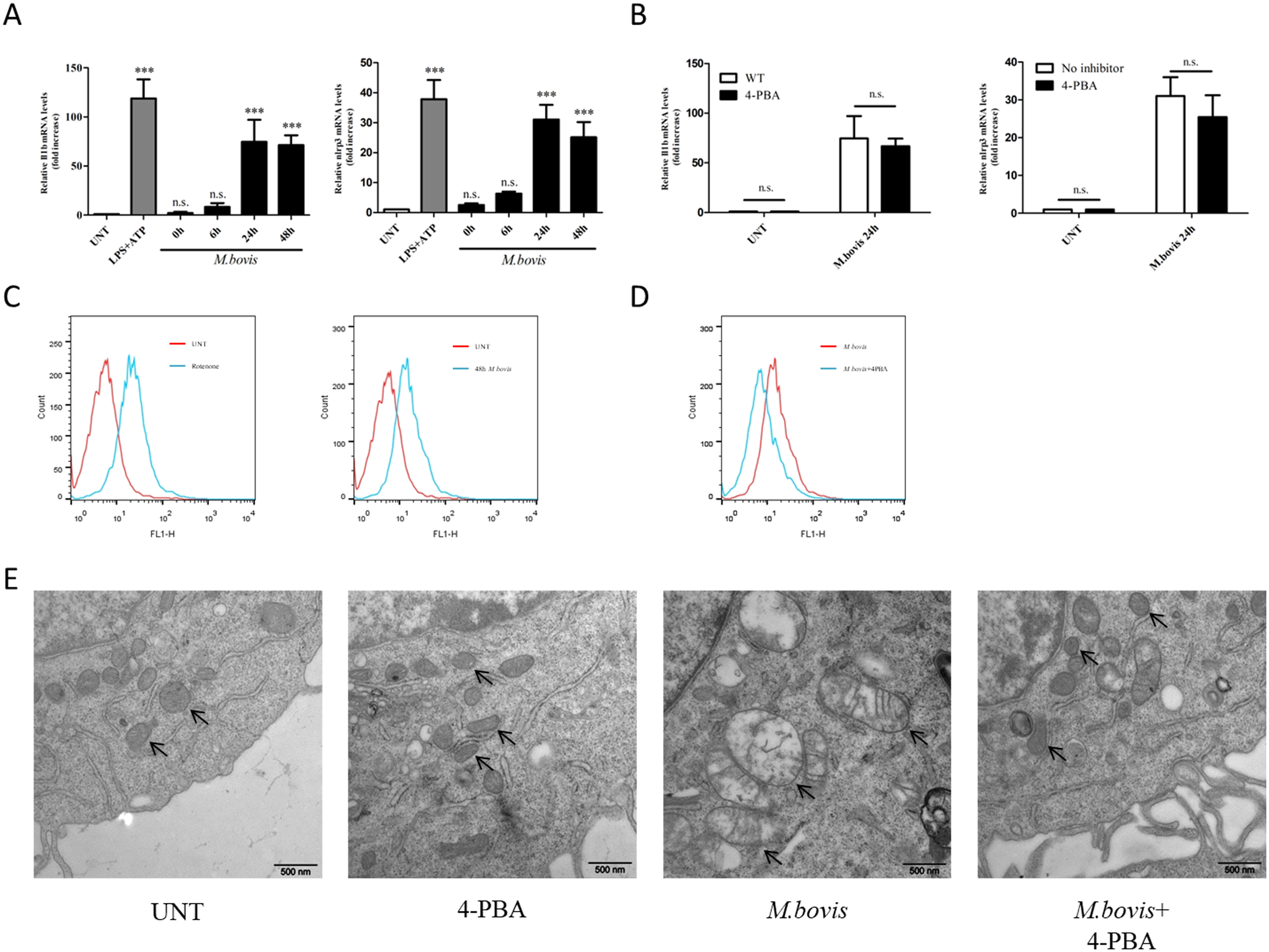

Supplement: Supplementary Figure 1 — ERS-induced mitochondrial dysfunction is involved in inflammasome activation. In support of Figure 2; (A) qPCR analysis of Il1b and Nlrp3 transcript in BMDMs infected with M. bovis (MOI 10). (B) qPCR analysis of Il1b and Nlrp3 transcript in BMDMs infected for 24 h with M. bovis (MOI 10) in the presence or absence of 4-PBA. (C) ROS production was measured at different time points by using flow cytometry in BMDMs infected with M. bovis (MOI 10). (D) ROS production was measured by flow cytometry in BMDMs infected for 48 h with M. bovis (MOI 10) in the presence or absence of 4-PBA. (E) TEM analysis of BMDMs infected with M. bovis (MOI 10) for 48 h in the presence or absence of 4-PBA. Arrows indicate mitochondria before and after infection. The data are representative of at least three independent experiments, each performed in triplicate. LPS+ATP, positive control for inflammasome activation, 200 ng/mL and 1 mM, respectively; 4-PBA, 4-phenyl butyric acid, ERS inhibitor, 5 mM; UNT, untreated; MOI, multiplicity of infection. [file Image_1.TIF]

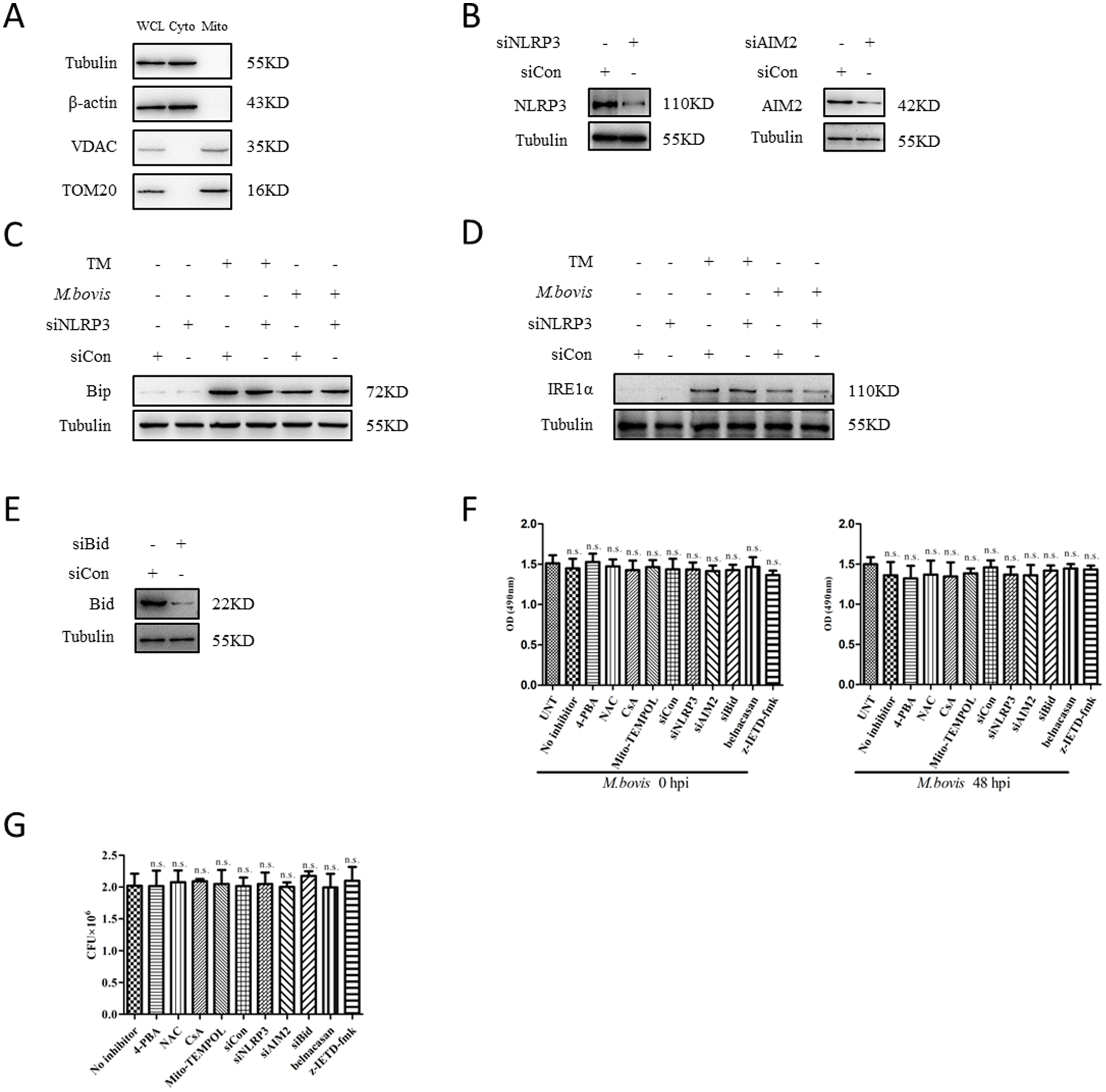

Supplement: Supplementary Figure 2 — (A) Immunoblot analysis of tubulin, β-actin (a cytosolic marker), TOM20, and VDAC (a mitochondrial marker) in whole cell lysate (WCL), the cytosolic fraction of cells (Cyto), and the mitochondrial fraction (Mito). (B) Immunoblot analysis of the expression NLRP3 and AIM2 in BMDMs transfected with control non-targeting siRNA (siCon), NLRP3-targeting siRNA (siNLRP3), or AIM2-targeting siRNA (siAIM2). (C) Immunoblot analysis of Bip in BMDMs transfected with control non-targeting siRNA or NLRP3 targeting siRNA and then infected for 24 h with M. bovis (MOI 10). (D) Immunoblot analysis of IRE1α in BMDMs transfected with siCon or siNLRP3 and then infected for 6 h with M. bovis (MOI 10). (E) Immunoblot analysis of the expression of Bid in BMDMs transfected with siCon or Bid -targeting siRNA (siBid). (F) Cell viability of BMDMs in the presence or absence of various inhibitors or siRNA. Inhibitors were added to cells 1 h prior to M. bovis infection (MOI 10). siRNA transfection medium was added to cells 48 h prior to M. bovis infection (MOI 10) and replaced with fresh medium 24 h prior to infection. After infection for 2 h, the inoculum was removed. The cells were washed with PBS and cultured at 37°C in an atmosphere of 5% CO2. At the indicated time points, the cell viability was measured. (G) Cell phagocytic capacity of BMDMs in the presence or absence of various inhibitors or siRNA. Inhibitors were added to cells 1 h prior to M. bovis infection (MOI 10). siRNA transfection medium was added to cells 48 h prior to M. bovis infection (MOI 10) and replaced with fresh medium 24 h prior to infection. After 2 h of infection, the inoculum was removed. Cells were washed with PBS and then lysed to enumerate intracellular CFU. UNT, untreated; 4-PBA, 4-phenyl butyric acid, ERS inhibitor, 5 mM; NAC, N-acety1-L-cysteine, the ROS scavenger, 5 mM; MitoTEMPOL, 4-hydroxy-2,2,6,6-tetramethylpiperidine-N-oxyl, mitochondria-targeted antioxidant agent, 500 μM; CsA, cyclosporine A, inhibitor of MPT [file Image_2.TIF]

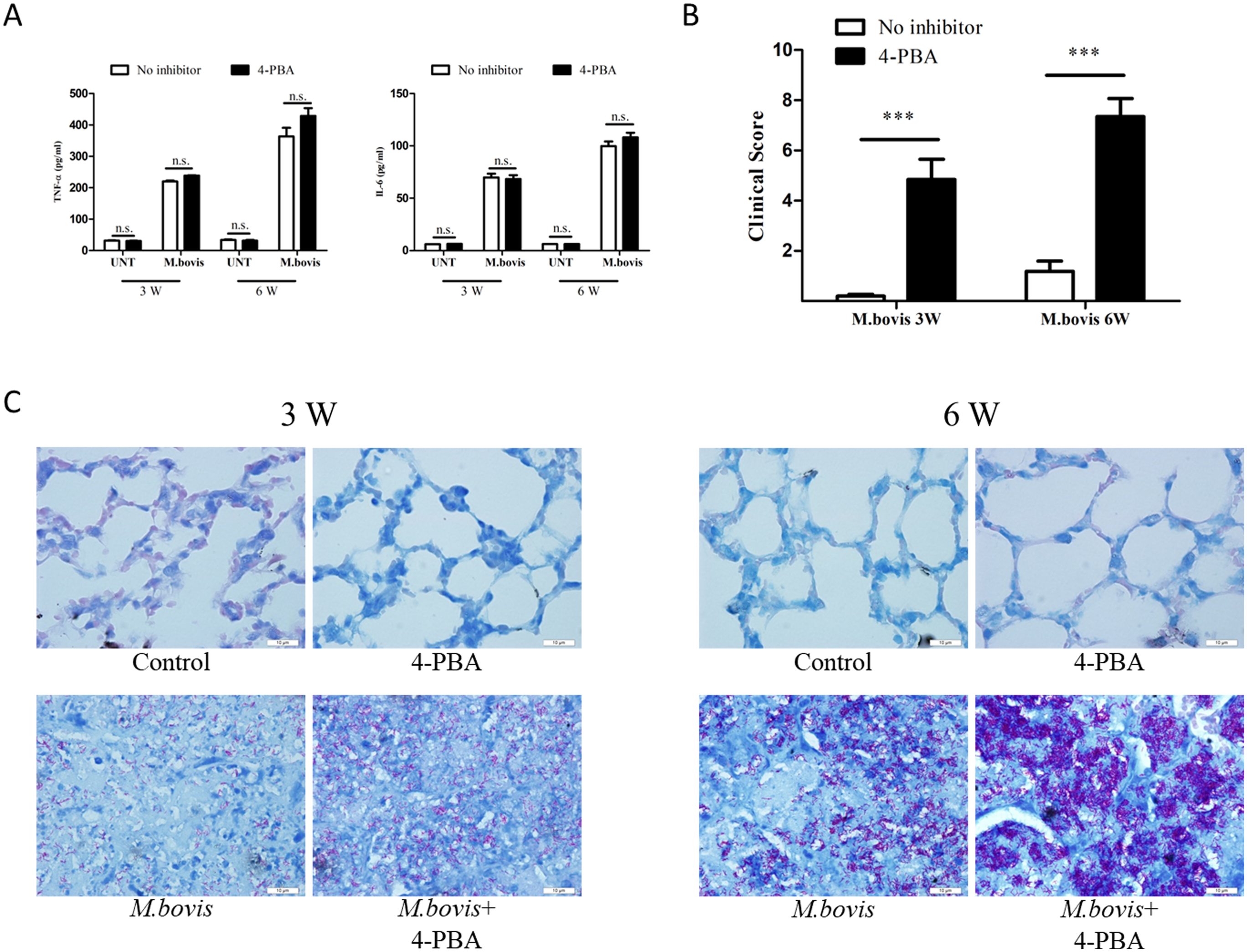

Supplement: Supplementary Figure 3 — ERS mediates M. bovis-induced inflammasome activation in vivo. Related to Figure 6. (A) ELISA analysis of serum TNF-α and IL-6 in mice treated with or without 4-PBA (18.6 mg/mouse/day) and infected with M. bovis (CFU 200) (n = 3). (B) Clinical scores of mice infected with M. bovis (CFU 200) for 3 weeks or 6 weeks in the presence or absence of 4-PBA (18.6 mg/mouse/day). (C) Bacterial burden (acid-fast staining) in the lung of mice infected with M. bovis (CFU 200) for 3 weeks or 6 weeks in the presence or absence of 4-PBA (18.6 mg/mouse/day). 4-PBA, 4-phenyl butyric acid, ERS inhibitor, 18.6 mg/mouse/day; CFU, colony forming units (n = 3). The data shown are the mean ± SD. ***P < 0.001, n.s., not significant. P-values were analyzed by using Student's t-test. (C) Scale bar = 10 μm. [file Image_3.TIF]
